# Growth Performance, Diet Digestibility, and Chemical Composition of Mealworm (*Tenebrio molitor* L.) Fed Agricultural By-Products

**DOI:** 10.3390/insects14100824

**Published:** 2023-10-20

**Authors:** Ana Montalbán, Silvia Martínez-Miró, Achille Schiavone, Josefa Madrid, Fuensanta Hernández

**Affiliations:** 1 Department of Animal Production, Faculty of Veterinary Science, Regional Campus of International Excellence “Mare Nostrum”, University of Murcia, Espinardo, 30100 Murcia, Spain; ana.montalban1@um.es (A.M.); alimen@um.es (J.M.); nutri@um.es (F.H.); 2 Department of Veterinary Sciences, University of Turin, 10095 Grugliasco, Italy; achille.schiavone@unito.it

**Keywords:** insect larvae, insect, protein source, bioconversion, grape pomace, tigernut pulp, broccoli

## Abstract

**Simple Summary:**

Insects are valuable alternatives to traditional protein sources for animal feed. Certain species have demonstrated that they can efficiently convert low-nutrient materials into quality body mass, making these species a viable protein and fat source. This study explores the effect of three agricultural by-products (broccoli by-product, tigernut pulp, and grape pomace) on the performance, digestive use, and proximate composition of *Tenebrio molitor* larvae. The growth and composition of the larvae were influenced by the type of by-product used, with the most protein-rich by-product resulting in larger larval biomass. However, diets based solely on a single by-product may penalize larval productivity and diet digestibility. The amino acid profiles of the insects were similar in all larvae, regardless of the diet, suggesting that these by-products can be used for insect rearing, achieving a sustainable production system aligning with the circular economy approach adopted globally.

**Abstract:**

Humanity’s growing demand for animal protein exceeds the capacity of traditional protein sources to support growing livestock production. Insects offer promising partial substitutes, converting low-nutritional quality materials into high-value biomass. Hence, the bioconversion ability of *Tenebrio molitor* larvae was assessed by using three types of agricultural by-products (broccoli by-product, tigernut pulp, and grape pomace) at different inclusion levels (0%, 25%, 50%, and 100%) in a carbohydrate-based diet. Ten diets were formulated to assess their impact on the growth, diet digestibility, and nutritional composition of the larvae. For each treatment, eight replicates were employed: five for the growth-performance-digestibility trial and three for the complementary test of uric acid determination. The growth was influenced by the type of diet administered. The broccoli by-product resulted in higher larvae weight and a better feed conversion ratio. However, diets based solely on a single by-product (100%) compromised the productivity and diet digestibility. The larvae changed their nutritional composition depending on the rearing substrate, although the amino acid profile remained consistent. In conclusion, the studied by-products have the potential for use in *T. molitor* rearing as part of the diet but not as the exclusive ingredients, indicating promising opportunities for using agricultural by-products in *T. molitor* rearing and production.

## 1. Introduction

Nowadays, new sources of protein are being sought to meet the high demand of mankind, while being more environmentally friendly. Climate change and other environmental issues are directly linked to the current food system. In the upcoming years, food-related greenhouse gas emissions are likely to increase significantly. As a result, to maintain safe planetary boundaries, future food systems must be able to produce enough food on less land, while also contributing to a carbon-neutral society [1]. Several studies have demonstrated that insect farming has numerous advantages in terms of production impact over other traditional sources of protein, leading to lower greenhouse gas emissions, needing less water and rearing land use, and having better conversion efficiency and reproductive rate, in addition to their ability to grow on substrates consisting of waste or by-products that can no longer be productive [2,3,4].

Some insect species are beginning to be considered as a food source of protein since their approval as a novel feed for intensive livestock in Europe (Commission Regulation (EU) 2021/1372 of 17 August 2021) [5] and can be used to partially substitute protein ingredients in compound feeds for livestock and aquaculture [6]. Edible insect species should be given consideration as potential sources of animal feed since insects offer a complete source of digestible proteins, essential amino acids [7] and fats, with profiles that may be modulated depending on the rearing substrate, among other factors [6].

On the other hand, the cost of the feeding substrate is critical to large-scale production since insects must be fed and temperatures and humidity must be maintained in insect rearing to optimize their growth, representing the most significant production expenses [6,8]. An important goal of the insect production industry is achieving cost-effective, efficient, and sustainable insect rearing for food and feed purposes. This entails the development of nutritionally optimized feeds that effectively support and maximize insect development and growth [9]. The use of waste and organic by-products is a potential and necessary cost reduction strategy for insect feed [10], as agricultural farming and agro-industrial systems produce significant quantities of waste materials that constitute valuable and unexplored resources suitable for insects’ substrates [2].

The yellow mealworm larvae (YML) have been recognized as one of the few candidate species exhibiting the potential for large-scale commercial production [9]. One of the key points is that the amino acid profile of the YML is similar to that of soybean, partially or completely meeting the demands of fish and livestock [11]. In this way, mealworm has been considered as a partial substitute for this raw material in monogastric animals, such as broilers [12,13], weaning pigs, and fish [13]. However, understanding the ideal nutritional composition for this insect species is crucial, as inadequate feed can result in extended production time and reduced nutrient use efficiency; therefore, using insects as a source of nutrition necessitates careful considerations to optimize production outcomes [14].

Against this background, there have been recent attempts to valorize various by-products of food or agricultural industry as substrates for maintaining the rearing of insects such as YML [15,16,17,18]. The mealworm is an omnivorous insect species [19,20] and has the ability to break down cellulose [20], a key component of fibrous wastes. Therefore, it may be possible to produce a high quantity of mealworms at lower economic costs, contributing to the revaluation of several agricultural industry by-products. Additionally, despite the known bioconversion capability of low-quality organic matter [21], topics such as the digestibility of by-products for mealworms remain understudied, requiring a better understanding for proper diet formulation. Considering the abovementioned importance, the aim of the current study was to examine the effects of feeding YML a variety of diets based on fibrous by-products of the Mediterranean area with different levels of inclusion and evaluate the influence of these diets on their performance and nutritional value.

## 2. Materials and Methods

### 2.1. Diet Preparation

The diets were prepared with by-products obtained from agro-industrial processes: brewer’s spent grains (waste grains from brewing, consisting of starch, germ, and hull residues from barley grains); bread remains; and three fibrous by-products: tigernut (*Cyperus esculentus*) pulp (the resulting product of grinding, soaking, and pressing for the artisanal production of tigernut milk); broccoli (*Brassica oleracea* var. italica) by-product, resulting from industrial discards; and grape pomace (seeds, skins, and some pulp remains from the wine industry). All by-products were dried at 60 °C to a constant weight (DM content: brewer´s spent grains, 249 g/kg; bread remains, 762 g/kg; tigernut pulp, 171 g/kg; broccoli by-product, 114 g/kg; and grape pomace, 435 g/kg), ground to a particle size of 1.0 mm for incorporation into the diets, and then dosed and mixed. The diets were administered in a mash format. Jellified water was available to the larvae as a humidity source.

The composition of the experimental diets is shown in Table 1. Ten by-product-based diets were designed: a control diet (CON) that contained bread remains (861 g/kg DM) and brewer´s spent grains (100 g/kg DM) and nine experimental diets in which each fibrous by-products (broccoli by-product (BB), (tigernut pulp (TP), and grape pomace (GP)) was included at 250 (25), 500 (50), or 961 g/kg DM (100). In the 25, 50, and 100 diets, the fibrous by-products replaced the bread remains and brewer´s spent grains. Additionally, a supplement containing minerals, vitamins, cholesterol, and linoleic acid [19] was added (39 g/kg DM) until 100% of the diet was completed.

### 2.2. Insect Rearing and Experimental Design

The *Tenebrio molitor* (L., 1785) (Coleoptera: Tenebrionidae) larvae were maintained in a pilot-scale insect-rearing room in the colony of Animal Nutrition Unit, Veterinary Faculty, University of Murcia, Spain. A total of 4000 larvae were collected in approximately the first half of their larval stage at an average weight of 17.46 ± 2.73 mg each. To prepare the larvae for the experiment, from egg hatching, they were kept in plastic boxes with a substrate similar to those used by other authors [22], comprising 80/20 oat flakes and brewer’s yeast (*Saccharomyces cerevisiae*). The larvae were kept under constant controlled temperature and humidity conditions (22 ± 3 °C and 40–50% RH) and natural photoperiod during the entire experimental period. To maintain the humidity, jellified water was administered in a round plastic container (3 cm diameter and 1 cm depth). The water was replenished periodically.

Five replicates per diet were intended for the performance-digestibility trial, and three replicates per diet for the uric acid complementary test for diet digestibility determination [15]. The replicates consisted of 50 larvae each one and were randomly distributed in plastic boxes (22.5 cm × 14.0 cm × 4.75 cm) with slits in the sides to allow air circulation. In performance-digestibility replicates, the feed was controlled and administered ad libitum (initially 1 g DM/replicate), and each replicate was monitored daily in terms of the feed available, number of live larvae, dead larvae, and pupae (both live and dead). On day 30 of the experiment, the larvae were weighed and frozen at −20 °C for further analysis. The litter, composed of refused diet and frass, was dried, weighed, and stored for determining the uric acid concentration and diet digestibility determination. In the replicates for uric acid determination, a one-time feed of 1.5 g DM was offered. The larvae were weighed every 15 days, and when weight growth ceased, we considered that the entire feed was consumed and frass constituted the entire litter. The frass was collected, weighed, and stored for determining the uric acid concentration.

### 2.3. Chemical Analysis

#### 2.3.1. Proximate Composition

The dry matter (DM) in the litter, frass, feeds, and by-products was determined after drying these for 48 h at 60 °C. The larvae were lyophilized to determine the DM. A laboratory mill was used to grind the larvae, by-products, and feed samples to 1.0 mm or 0.5 mm when necessary for calculating the starch and amino acid content (RETSCH ZM 200 Ultra Centrifugal Mill; RETSCH, Hann, Germany). The larvae and diets were chemically analyzed according to the procedures specified by the Association of Official Analytical Chemists (AOAC) [23] for crude protein (CP) (method 2001.11) and ether extract (EE) (method 920.39). Ashes were determined by ashing samples at 550 °C for 4 h in a muffle furnace (Hobersal HK-11, Barcelona, Spain). The starch content was determined polarimetrically in the by-products and feed samples following the official Spanish analytical method (RD 2257/1994) [24]). In addition, neutral detergent fiber (NDF), acid detergent fiber (ADF), and lignin acid detergent (LAD) were analyzed according to the procedures described by Van Soest et al. [25].

#### 2.3.2. Uric Acid

To determine the uric acid concentration, the UV–Vis spectrophotometry method mentioned by Marquardt [26] was used (UNI-CAN UV–Vis Spectrometry, Helios Gamma, Loughborough, UK). A uric acid concentration of 50 mg of either the rejected substrate or the pure frass was determined by spectrophotometry after these were incubated for 1 h in 100 mL of glycine buffer solution at pH 9.3.

#### 2.3.3. Amino Acid Analysis

The quantity of amino acids (AAs) in the larvae was determined in accordance with the procedure described by Madrid et al. [27]. Dried samples ground to 0.5 mm were hydrolyzed in 6 N HCl at 112 °C under a nitrogen atmosphere for 22 h. After derivatization, the levels of AA in the hydrolysate were determined by HPLC on a Waters Alliance System equipped with a Waters 1525 Binary HPLC pump, a Waters 2707 autosampler, and a Waters 2475 multi-fluorescence detector (Waters, Milford, MA, USA).

### 2.4. Performances and Digestibility Calculation

Based on the amount of uric acid in the pure frass, the total amount of refusal feed and excreta was determined [15]. Then, we calculated the feed intake and monitored the weight to calculate the feed conversion ratio (FCR) according to the following formula:

FCR = Feed intake (g DM)/Weight gained (g DM)

The digestibility was calculated weighing the total excreta from each replicate, assuming that uric acid excretion was constant between replicates for each by-product diet. The apparent total tract digestibility coefficient for DM (ATTD) was determined as follows:

ATTD% = ((DM ingested − DM excreted)/(DM ingested)) × 100

### 2.5. Statistical Analysis

Data were analyzed using the general linear model (GLM) procedure (SPSS 28 version). The statistical model for analysis of variance was as follows:

Y _ij_ = μ + LI _i_ + TB _j_ + (LI × TB) _ij_ + E _ij_

where Y _ij_ is a dependent variable, μ is the overall mean, LI is the effect of the level of inclusion (fixed effect), TB is the effect of the type of by-product (fixed effect), (LI × TB) _ij_ is the interaction between by-product inclusion level and by-product type, and E is the random residual error. A Tukey test was used to separate means. *p*-values of ≤0.05 were regarded as significant.

## 3. Results

### 3.1. Performance and Digestibility

**Figure 1:**
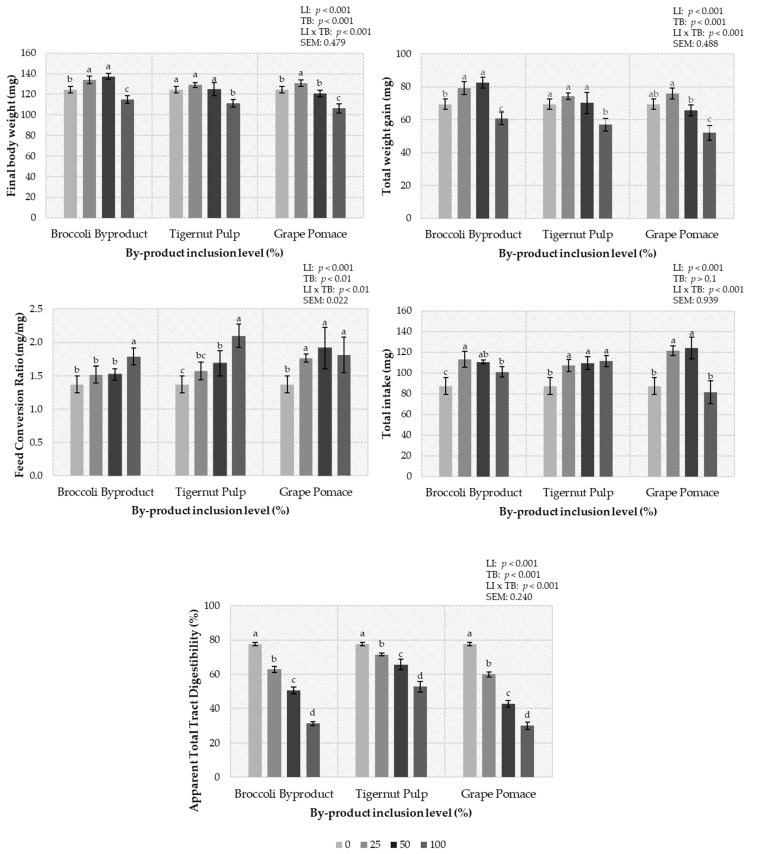
Effect of the inclusion level (0, 25, 50, and 100%) of the broccoli by-product (BB), tigernut pulp (TP), and grape pomace (GP) in the diets of *Tenebrio molitor* larvae on the final body weight, the total weight gain, the feed conversion ratio, the total intake, and apparent total tract digestibility. (*n* = 5). *p*-values are provided for BB, TP, and GP diets and for LI (level of inclusion), TB (type of by-product), and LI × TB (interaction between LI and TB). Mean ± standard deviation. ^abcd^ (*p* < 0.05).

The results for the productive performance of the YML are shown in Figure 1. At the end of the experimental period, among the larvae that were fed the three by-product diets, those that were fed the broccoli by-product diets were the heaviest (127.68 mg for BB, 122.38 mg for TP, and 120.56 mg for GP) (*p* < 0.001). The total weight gain was affected by the type of by-product and its inclusion levels (*p* < 0.001). However, for all diets, a major level of inclusion (100%) penalized the larval growth (*p* < 0.001). Also, an interaction was identified (*p* < 0.001). Inclusion levels of 25% (25BB) and 50% (50BB) of BB appear to promote larval body weight compared to the 0% diet (CON) and the 100% (100BB) diet (79.21, 82.58, 69.46, and 60.82 mg for 25BB, 50BB, CON, and 100BB, respectively). Regarding TP diets, similar total weight gains were found for 0TP, 25TP, and 50TP (69.46, 74.31, and 70.18 mg for 0TP, 25TP, and 50TP, respectively) and these were higher than that for 100TP (56.97 mg). However, for the GP diets, the highest final weight gain was for the 25GP diet, similar to that for the 0GP diet. The larvae fed the 50GP diet had a lower growth than those fed the 25GP diet but a similar growth to those fed the 0GP diet. Finally, the worst gain was observed in the larvae fed the 100GP diets (69.46, 75.90, 65.66, and 52.03 mg for 0GP, 25GP, 50GP, and 100GP, respectively).

The total intake was not affected by the type of by-products (*p* > 0.05). However, overall, the increase in the inclusion levels of by-products by 25% and 50% resulted in a noteworthy increase in total feed intake, followed by the diets that incorporated 100% and 0% of by-products (113.87, 114.60, 97.88, and 87.19 mg for 25, 50, 100, and 0%, respectively) (*p* < 0.001). An interaction was found in this parameter as well (*p* < 0.001). While in the BB diets, the highest intakes were observed in the 25BB and 50BB diets, followed by the 100BB and CON diets (87.19, 113.14, 110.44, and 100.94 mg for CON, 25BB, 50BB, and 100BB, respectively) (*p* < 0.001), in the TP diets, the highest intakes were obtained in the 100TP, 50TP, and 25TP diets, which were significantly higher that the intake observed in the CON diet (87.19,107.02, 109.48, and 111.22 mg for CON, 25TP, 50TP, and 100TP, respectively) (*p* < 0.001); and in the GP diets, the highest intakes were observed in the 50GP and 25GP diets, which were significantly higher than the intakes in the CON and 100GP diets (87.19, 121.46, 123.87, and 81.47 for CON, 25GP, 50GP, and 100GP, respectively).

The type of by-product influenced the FCR (*p* < 0.01), with no statistical differences between the TP and GP diets but with the best conversion ratio in the diets with BB (1.68, 1.72, and 1.55 for TP, GP, and BB, respectively). Moreover, the FCR worsened, in general, as the level of inclusion of the by-product increased (1.37, 1.62, 1.71, and 1.90 for 0%, 25%, 50%, and 100%, respectively) (*p* < 0.001). An interaction was also found (*p* < 0.01). In other words, regarding the BB diets, the CON, 25BB, and 50BB diets had no differences in terms of conversion ratio and the 100BB diet had the worst ratio (1.37, 1.52, 1.52, and 1.79 for CON, 25BB, 50BB, and 100BB, respectively). Furthermore, the CON diet obtained the best FCR in the TP diets, the 100TP diet (2.10) having the worst FCR. When the GP diets were compared, the 25GP, 50GP, and 100GP diets did not show statistical differences in terms of FCR (*p* > 0.05), the CON diet having the best FCR (1.37, 1.76, 1.92, and 1.81 for CON, 25GP, 50GP, and 100GP, respectively).

The ATTD of dry matter was affected by both factors, the type of by-product and the level of inclusion (*p* < 0.001). Statistical differences were found between the three types of by-products, with the diets containing TP having the highest levels of digestibility, followed by the BB diets (exhibiting intermediate results), while the GP diets were found to be the least digestible (66.88, 55.58, and 52.60 for TP, BB, and GP, respectively) (*p* < 0.001). Moreover, the ATTD worsened in all the experimental diets as the level of inclusion of by-product increased (77.66, 64.73, 52.48, and 38.03 for 0, 25, 50, and 100%, respectively) (*p* < 0.001). An interaction was also noticed (*p* < 0.001), although the ATTD dry matter decreased as the level of by-product inclusion in all the diets was increased; the decrease was greater for BB and GP diets.

### 3.2. Chemical Composition of Insects

**Figure 2:**
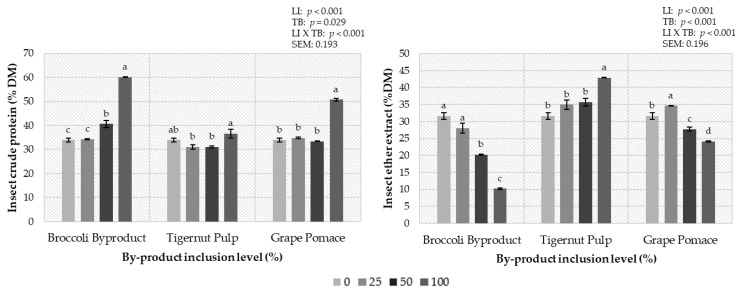
Effect of increasing level (0, 25, 50, and 100%) of the broccoli by-product (BB), tigernut pulp (TP), and grape pomace (GP) in the diets of *Tenebrio molitor* larvae on crude protein and ether extract insect composition (*n* = 5). *p*-values are provided for LI (level of inclusion), TB (type of by-product), and LI × TB (interaction between LI and TB). Mean ± standard deviation. ^abcd^ (*p* < 0.05).

The chemical composition of insects in terms of crude protein and ether extract are shown in Figure 2. Statistical differences were found between the protein content and ether extract, depending on the type of by-product and the level of incorporation of the by-product used. Regarding the type of by-product, the larvae reared on BB diets had the highest crude protein level, followed by those reared on TP and GP diets (42.22, 36.68, and 34.65% for BB, TP, and GP, respectively) (*p* < 0.001). Regarding the level of inclusion, in this study, larvae with the highest protein concentration were obtained when the by-product inclusion level was 100% (*p* < 0.001). However, the ether extract followed the inverted tendency in terms of the type of by-product. The larvae reared on the GP diets had the highest ether extract content, followed by those on the TP diets and those on the BB diets (34.21, 31.58, and 22.51% for GP, TP, and BB, respectively) (*p* < 0.001). Regarding inclusion level, the larvae with more ether extract content were those reared on a diet incorporating 0% and 25% of the by-product and the ether extract content worsened as the level of inclusion increased (*p* < 0.001). An interaction between these two parameters, type of by-products and inclusion level, was found (*p* < 0.001). Regarding the larvae reared on a BB diet, the highest protein content was found in the larvae reared on a 100BB diet (60.09%) and the lowest protein content was found in those reared on a CON diet (33.91%) (*p* < 0.001). An inverse tendency was observed in the ether extract content of the insects, with those reared on the 0% diet showing the highest ether extract (31.58%) and those reared on the 100BB diet showing the lowest ether extract (10.21%) (*p* < 0.001). The TP by-product maintained the most consistent content in both parameters independently of the inclusion level, with the highest protein content found in the larvae reared on a 100TP diet (36.55%) compared to those on the 25TP diet and the 50TP diet (31.01 and 31.09% for 25TP, and 50TP, respectively) (*p <* 0.05). The highest fat content was found in the 100TP larvae and the lowest was found in the CON larvae (42.90% and 31.58 for 100TP and CON, respectively) (*p* < 0.01). The protein content of the larvae reared on the GP diets had no statistical differences when the inclusion levels were 0%, 25%, and 50%, but there was a remarkable increase when the inclusion level was increased to 100%, reaching a value of 50.71% (*p* < 0.001). This last inclusion level also provided the least fatted larvae (24.13%) (*p* < 0.001). The highest fat content was found in the larvae reared on a 25GP diet.

The amino acid profiles of the larvae reared on the different diets did not differ except in alanine and proline content (Table 2), which were influenced by the type of by-product, being lower in those larvae reared on BB diets (*p* < 0.05 and 0.01 for alanine and proline, respectively). The level of inclusion of by-product did not affect the amino acid profile of the larvae (*p* > 0.05). There was no interaction between the type of by-product and the level of inclusion.

## 4. Discussion

The fact that insects are rich in protein and fat is the main reason for the focus on insect use in animal feed. Furthermore, due to the bioconversion capability of organic by-products, their use as feed represents environmental advantages within the context of the circular economy the world is pursuing. Whether these by-products are useful depends upon how efficiently the production animal perform. Thus, due to the ability of insects to convert feed into body mass [28], a great variety of by-products can be used in insect diets, which is reflected in several studies that are exploring agricultural side streams as feed for rearing YML, as reviewed by Van Peer et al. [29].

In the present study, three agricultural by-products (BB, TP, and GP) were used at different inclusion levels (from 0% to 100%) to investigate their effects on the growth, ATTD of DM, and proximate composition of YML. The diets with the highest protein content by-product (BB) resulted in the highest larval weight, which is in accordance with the findings of Rumbos et al. [30] who found that the larvae fed the lupin by-product, with remarkable protein content, exhibited higher final weight compared to those given other dietary treatments with less protein content. The same finding was made by Montalbán et al. [31] in a study where Mediterranean by-products were also used. Similarly, in a recent study, Ruschioni et al. [21] assessed different levels of a by-product from the olive oil industry (olive pomace), finding that the larvae with higher weights were those fed more protein-rich diets. Additionally, van Broekhoven et al. [15], using high amounts of yeast-derived protein, showed similar results. However, it should be noted that in the present study, when the level of the BB by-product was 100%, the development of the larvae decreased (total weight gain). Kröncke and Benning [32] pointed out a negative correlation between the protein content of the diets up to 20% and the individual larval weight, which could justify the lower development observed in the larvae reared on a 100% BB diet.

For by-products with higher fiber content (TP and GP), a similar result was observed regarding larval weight gain when the level of inclusion was at a maximum. Thus, the incorporation level of 100% of the by-product penalized the development of the larvae. This behavior has been highlighted by Rumbos et al. [33], indicating that when certain Greek-origin by-products were used as the only ingredient in their diets, the YML exhibited lower growth compared to the larvae given a control diet composed of wheat bran. Other authors, such as Li et al. [34] and Morales-Ramos et al. [35], noted that high levels of fiber in feed could have a negative impact on the growth of YML, probably due to a form of nutritional stress.

The variability in the feed conversion ratio found in the literature is significant and highly dependent on the composition of the administered diet [15,28,30]. This study suggests that the experimental diets evaluated exhibited an increase in this parameter compared to the CON diet. Thus, the worsening of the FCR is associated with feed intake and weight gain. This behavior can be attributed to the adaptation of YML to cereal-based diets, as this species is characterized by infesting stored grains and cereals [36]. In this study, the most protein-rich by-product (BB) showed a lower FCR compared to the other two diets with higher fat content (TP and GP). However, when any of these by-products is administered at 100%, the FCR worsens drastically. Kröncke and Benning [32] pointed out that the FCR was better in diets with lower protein content than in those with higher protein levels, being more efficient. In our case, this behavior was observed only in the larvae reared on BB-based diets. According to other authors [37], this could be explained by the decreased efficiency of the larvae in using protein once they have reached their requirements for growth. However, Morales-Ramos et al. [35] found that the intake of fiber negatively affected feed assimilation, feed conversion, and biomass gain (consistent with our results regarding TP and GP by-products). Li et al. [34] stated that the optimal fiber levels for YML would be below 10% of diet incorporation, which could explain the worsening in the FCR in all the experimental diets we administered.

Regarding the digestibility of the three by-product diets administered to YML, TP exhibited the highest digestibility. This could be attributed to the higher levels of fat and starch in the TP diets compared to the other experimental diets. Additionally, as the level of by-product increased, digestibility worsened in all cases, which could be justified by the increase in the fiber level, contributing to less digestible rations, as previously mentioned by other authors [31,35]. Although YML can fulfill all essential nutritional requirements when consuming wheat bran [38], research studies have demonstrated that adding supplementary ingredients can enhance larval survival and development time [15,17,39].

With respect to the survival rate, this study revealed that diets based on BB, TP, and GP did not result in higher larval mortality. Irrespective of the diet, the maximum mortality rate observed was consistently below 5% for all larvae. These obtained mortality values were, in general, lower than those reported in the recently published literature, although this is a parameter that depends on the type of diet administered [17,21,36]. However, some studies show even lower mortalities [32], suggesting that dietary amino acids have a significant impact on the life cycle of YML, directly influencing their growth, weight gain, and survival.

*T. molitor* larvae has a high protein content. The larvae under study displayed substantial variability in protein proportion, ranging from a minimum of 31.01% (25TP) to a maximum of 60.09% (100BB). This serves as evidence of the remarkable capability of YML to modify its nutritional composition in response to dietary factors [40]. In general, diets with the highest protein level by-product (BB) led to larvae with the highest protein content, which could suggest a positive correlation between the protein content of the diets and the larvae [28,31,32]. However, in the present experience, all the studied by-products resulted in larvae with higher protein content when incorporated at a 100%, despite the fact that the TP and GP diets had a lower protein content than BB diets. Similar results were highlighted by Kotsou et al. [41], who used a high-fiber-content agricultural waste (orange albedo) diet, which also has a low protein content, showing that as the level of by-product increases in the diet, the protein content that reaches the larvae increases. This is also in contrast with the results reported by Ruschioni et al. [21], who used a high-fiber-content by-product (olive pomace) in different proportions and found the larvae with the highest protein content in those reared on a diet with intermediate protein and fat levels. In other words, it was neither the diet with the highest protein content nor the one with the maximum inclusion of the by-product but rather an intermediate incorporation of it. Therefore, factors beyond dietary protein and fiber can influence larvae composition.

YML are recognized as a sustainable protein source alternative to soybean meal or fishmeal due to their rich protein content and amino acid profile. Among the indispensable amino acids, Leu, Val, and Lys are found in the highest abundance in YML [13]. Kröncke and Benning [32] found that the content of alanine in the larvae varied on the basis of the type of diet administered, as highlighted by the present study. In the present study, the AA content of the larvae was not affected by the type of by-product except in the case of alanine and proline content, which were lower in the larvae reared on BB-based diets, despite these diets having the highest protein content. This further supports the idea that the amino acid content of the diet is not a key factor in the final chemical composition of the larvae, contrary to that expressed by other authors [42].

Similar to other animal species, YML have the capability to store fat as an energy source. Hence, this insect species should be considered not only as a protein source but also as a valuable source of dietary fat. For this reason, it is important to characterize the fat content of YML depending on these experimental diets. Some authors who included high-fiber by-products such as olive pomace have indicated that the fat content of the larvae does not correlate with the fat content of the diet [21], contrary to our findings, where both the type of by-product and its inclusion level influenced the fat content of the larvae. Other authors have found correlations, such as Kotsou et al. [41], who supplemented the YML diet with orange albedo, another highly fibrous by-product, observing that higher levels of this by-product led to lower fat levels in the larvae, attributing this to the low fat content of the albedo. In our study, the same general behavior was observed in the larvae administered the BB and GP diets but the opposite was observed in the TP-diet-reared larvae. Although the TP diet too had a high percentage of fiber, the increase in the fat content of these larvae may be attributed to the high level of fat content in this by-product. However, in a recent study, Bordiean et al. [42] concluded that lower levels of dietary protein resulted in a higher fat content in the larvae, which aligns with our findings in the larvae raised on BB and TP diets.

Some authors have pointed out that diets characterized by elevated levels of protein and fat exhibit notable enhancements in various biological parameters compared to diets featuring high carbohydrate content [39]. In the present study, diets incorporating by-products with higher protein levels led to greater larval weight and improved FCRs. However, diets containing higher fiber content resulted in elevated fat content and higher FCRs. Nevertheless, the larvae maintained a consistent amino acid profile irrespective of the dietary substrate.

According to our findings, it can be deduced that the use of these by-products did not inhibit larval growth but in fact, in most cases, stimulated their growth. So, these by-products could be used as growth substrates for YML, aligning with the principles of a circular economy. However, they should not be used as the exclusive ingredient in the diet. Further studies are needed to gain a more precise understanding of the nutritional requirements of the larvae of this insect species, which will enable us to fully develop its potential as a novel feed source.

## 5. Conclusions

This study shows that the growth, diet digestibility, and proximate composition of *Tenebrio molitor* larvae were affected by the type of diet and the level of inclusion of by-products employed. The broccoli by-product, with the highest protein content, yielded better larval weight and improved the feed conversion ratio more than the other two by-products, GP and TP. However, diets exclusively composed of one by-product negatively impacted productivity and diet digestibility. The proximate composition of the larvae changed depending on the rearing substrate. In general, diets richer in protein led to insects with a higher protein content, while diets with a higher fiber content led to insects with a reduced protein content. Nonetheless, the amino acid profile remained stable regardless of the diet. These findings suggest the potential of these by-products to serve as a viable rearing substrate for *Tenebrio molitor* larvae, contributing to their revalorization within the context of sustainability and circular economy approaches.

## Figures and Tables

**Table 1:** Composition of the experimental diets provided to *Tenebrio molitor* larvae.

Ingredient (g/kg DM)	CON ^a^	25BB	50BB	100BB	25TP	50TP	100TP	25GP	50GP	100GP
Bread remains	861	611	361	-	611	361	-	611	361	-
Brewer´s spent grains	100	100	100	-	100	100	-	100	100	-
Supplement ^b^	39	39	39	39	39	39	39	39	39	39
Tigernut pulp	-	-	-	-	250	500	961	-	-	-
Broccoli by-product		250	500	961	-	-	-	-	-	-
Grape pomace								250	500	961
Chemical composition (g/kg DM)										
Ash	22	45	68	115	19	15	9	30	38	54
Crude protein	143	160	178	205	123	104	56	138	133	114
Starch	680	484	287	0	530	382	191	484	287	0
Ether extract	11	20	30	41	57	102	186	35	60	101
Neutral detergent fiber	91	125	160	191	214	338	548	196	302	475
Acid detergent fiber	20	56	91	146	79	137	238	105	190	345
Lignin acid detergent	3.1	5	8	11	14	24	44	63	122	240

^a^ CON control diet; BB, broccoli by-product; TP, tigernut pulp; GP, grape pomace. ^b^ Provided the following per kilogram of diet: linoleic acid, 5000 mg; cholesterol, 5000 mg; ascorbic acid, 3000 mg; choline, 1250 mg; inositol, 250 mg; niacin, 100 mg; D-pantothenic acid, 50 mg; riboflavin, 25 mg; folic acid, 25 mg; thiamine, 25 mg; D-biotin, 1 mg; pyridoxine, 25 mg; potassium phosphate monobasic, 7440 mg; calcium carbonate, 5040 mg; tricalcium phosphate, 3576 mg; potassium chloride, 2880 mg; sodium chloride, 2520 mg; magnesium sulfate.7H_2_O, 2160 mg; ferric phosphate, 352 mg; sodium fluoride, 13,68 mg; copper sulfate.5H_2_O, 9.36 mg; manganese sulfate.H_2_O, 4.8 mg; potassium aluminum sulfate, 2.16 mg; potassium iodide, 1.2 mg.

**Table 2:** Effects of the diets on the amino acid composition of *Tenebrio molitor* larvae (expressed as a crude protein percentage) (*n* = 5).

Level of Incorporation (LI)					Type of By-Product (TB)				*p*-Value		
	0	25	50	100	BB	TP	GP	SEM ^1^	LI	TB	LI × TB ^2^
Cys	0.79	0.77	0.78	0.76	0.76	0.78	0.78	0.030	0.150	0.430	0.454
Asp	8.01	8.18	8.08	8.45	8.15	8.18	8.22	0.298	0.247	0.750	0.948
Ser	5.26	5.23	5.49	5.28	5.26	5.35	5.32	0.115	0.290	0.855	0.994
Glu	9.72	12.45	12.06	12.60	12.02	11.25	11.85	0.438	0.123	0.754	0.951
Gly	6.36	5.83	5.07	5.74	5.97	5.45	5.83	0.253	0.392	0.694	0.734
His	4.00	3.12	3.38	3.26	3.51	3.58	3.23	0.120	0.102	0.471	0.393
Arg	6.91	5.92	6.51	6.11	6.48	6.53	6.08	0.154	0.165	0.450	0.591
Thr	4.64	4.23	4.46	4.28	4.47	4.44	4.29	0.109	0.142	0.612	0.884
Ala	8.13	8.29	7.82	8.45	7.26 ^b^	8.50 ^a^	8.76^a^	0.186	0.676	0.014	0.024
Met	2.22	2.40	2.26	2.16	2.36	2.14	2.28	0.148	0.945	0.831	0.833
Pro	7.04	7.82	7.42	7.43	6.81 ^b^	7.77 ^a^	7.69 ^a^	0.112	0.166	0.008	0.522
Tyr	8.72	7.30	8.08	6.78	8.07	8.02	7.07	0.475	0.515	0.639	0.524
Val	6.33	6.74	6.72	6.76	6.54	6.71	6.67	0.080	0.221	0.677	0.914
Lys	5.16	6.17	5.81	6.40	5.93	5.50	6.23	0.222	0.270	0.426	0.603
Ile	4.60	4.81	4.85	4.73	4.69	4.77	4.78	0.085	0.744	0.908	0.961
Leu	7.63	7.25	7.40	7.33	7.42	7.42	7.36	0.048	0.077	0.853	0.684
Phe	4.75	3.51	3.82	3.49	4.17	3.99	3.51	0.179	0.091	0.333	0.507

^1^ SEM, Standard error of the mean. ^2^ LI × TB, Interaction (type of by-product x level of inclusion). ^ab^, means values followed by different letters in the same row are different (*p* < 0.05).

## Data Availability

The data presented in this study are available on request from the corresponding author.
